# Journal of Materia Medica and Pharmaceutic Formulary

**Published:** 1858-05

**Authors:** 


### Journal of Materia Medica and Pharmaceutic Formulary.

We have received from Messrs. Tilden & Co., New Lebanon, Columbia County, N. Y., the Journal of Materia Medica and Pharmaceutic Formulary, the object of which will be found stated in the article below, taken from its pages:

"To Physicians and Apothecaries.—It is with much pleasure we again issue our Journal, enlarged in size and with a new title. The encouragement which our plan has received from the medical profession has suggested this change. We shall give a greater amount of original and selected matter, and make it a practical Journal of Materia Medica and Pharmaceutic Formulary. Each number will contain one or more articles upon those plants which are directed for medical use, embracing a botanical description, its natural history, and chemical and medical characteristics, with a circumstantial detail of their respective virtues and the diseases in which they have been most successfully employed by different authors—communications from physicians, notices of new preparations, all discoveries of new properties in remedies now in use, articles from Medical and Pharmaceutical Journals of interest to the profession, and particularly formulas for pharmaceutic preparations, valuable to the physician and apothecary.

Practitioners will always find its columns open to all communications upon subjects of interest to the profession, and are solicited to communicate their personal observations and experience in the use of any plants or remedies new or interesting.

We shall expect to issue the paper every month, and our terms will be 25 cents per year, or for 12 numbers, which may be remitted in postage stamps. All old subscribers will be regularly supplied.

